# Effect of Varying Curing Conditions on the Strength of Biopolymer Modified Sand

**DOI:** 10.3390/polym15071678

**Published:** 2023-03-28

**Authors:** Kehinde Lemboye, Abdullah Almajed

**Affiliations:** 1 Department of Civil Engineering, College of Engineering, King Saud University, Riyadh 11421, Saudi Arabia; 2 School of Civil and Mechanical Engineering, Curtin University, Bentley, WA 6102, Australia

**Keywords:** acacia, biopolymer, properties, gelling, pectin, sodium alginate, soil stabilization unconfined compressive strength

## Abstract

Recently, the improvement of the engineering properties of soil has been centered on using sustainable and eco-friendly materials. This study investigates the efficacy of three biopolymers: Acacia, sodium alginate, and pectin, on the unconfined compressive strength (UCS) of dune sand. The UCS test measured the effects of the biopolymer type and concentration, curing intervals and temperature, and moisture loss. The changes in the morphology caused by the biopolymer addition were examined via scanning electron microscopy (SEM). Results indicate that the UCS of the biopolymer-modified sand increased with biopolymer concentration and curing intervals. Varying the curing temperature from 25–110 °C, slightly affected the strength of the acacia-modified sand specimen, increased that of the sodium alginate-modified sand specimen up to a temperature of 85 °C, and continued to decrease that of the pectin-modified sand specimen as the temperature was increased from 25 to 110 °C. The SEM images indicated that the biopolymer’s presence within the sand pores significantly contributed to the strength. Bond decomposition occurs at temperatures greater than 110 °C for sodium alginate and pectin-modified sands, whereas bonds remain stable at higher temperatures for the acacia-modified sand. In conclusion, all three biopolymers show potential as robust and economic dune stabilisers.

## 1. Introduction

Soil properties such as strength, volume stability, durability, compressibility, and permeability are essential to geotechnical applications, from dune stabilisation to foundation design [1]. Traditional methods for improving the geotechnical properties of soil include mechanical and chemical techniques. Conventional methods for chemical stabilisation, which employ materials such as cement, lime, fly ash, cement kiln dust, and blast-furnace slag, are the oldest and have gained popularity among researchers [2,3,4,5,6,7,8,9,10,11,12,13,14,15,16,17,18,19,20,21]. These materials either aid compaction, repel water, or bind to the soil particles when incorporated into the soil matrix. Soil treated with chemicals, except sodium silicate, is often prone to environmental hazards [22]. Cement and lime can change the pH of the soil, pollute the groundwater, and pose a threat to human health. The cement production industry is one of the most significant sources of carbon dioxide (CO_2_) emissions, accounting for approximately 8% of global emissions [23]. About 60% of these emissions result from chemical reactions, whereas the remaining 40% are due to the combustion of fossil fuels during cement production [24]. Attempts to reduce the massive carbon footprint of cement production have given rise to adopting nontraditional stabilisation agents, such as waste materials, lignosulfonate, epoxy resins, polymers, geopolymers, and biopolymers [25,26,27,28,29,30,31,32,33]. These additives are cost-and-time-effective alternatives to conventional materials. 

Biopolymers have many applications in various fields, including water treatment, medicine, paper production, energy, oil exploration, and textile and food processing. They are produced from renewable resources and are biodegradable and non-toxic, making them suitable for use in geotechnical engineering. In this field, biopolymers have been reported to improve soil properties [22,34,35,36,37,38,39,40,41,42,43,44,45,46]. Taytak et al. (2012) [46] reported an increase in the maximum dry density and a reduction in the permeability of the bentonite–kaolin–sand mixture with an increase in the biopolymer concentration. Latifi et al. (2016) [43] investigated the effect of curing time and biopolymer concentration on the shear strength of xanthan-gum-modified organic peat. The curing periods were 3, 7, 28, and 90 days, with biopolymer concentrations of 0.5%, 1.0%, 1.5%, and 2.0%. The cohesion, strength, and friction angle increased significantly with increases in curing time during the first 28 days at an optimum concentration of 2%. Qureshi et al. (2017) [45] investigated the UCS and unconsolidated undrained shear strength of sand modified with xanthan gum (concentrations of 0%, 1%, 2%, 3%, and 5%) at confining pressures of 50, 100, and 150 kPa. This study reported an optimal concentration of 2% to achieve maximum increases in the UCS strength. However, strength decreased at higher concentrations. A study by Fatehi et al. (2019) [38] used sodium alginate in concentrations of up to 5% with different curing times and temperatures. The results indicated a correlation between UCS increases and biopolymer concentration. The strength of the sand modified with a biopolymer concentration of 2% increased rapidly for the first 14 days of curing, with a slight increase observed after 28 days. The strength increased as the curing temperature rose to 45 °C and decreased at temperatures higher than 45 °C after 14 days.

Most geotechnical engineering studies have focused on using xanthan and guar gums to improve soil mechanical properties. This research investigates the effects of acacia, sodium alginate, and pectin on soil properties. Laboratory tests, including unconfined UCS and SEM techniques, used different concentrations, curing periods, and temperatures to provide insights into the mechanical properties of the modified sand.

## 2. Materials and Methods

### 2.1. Soil

**Figure 1:**
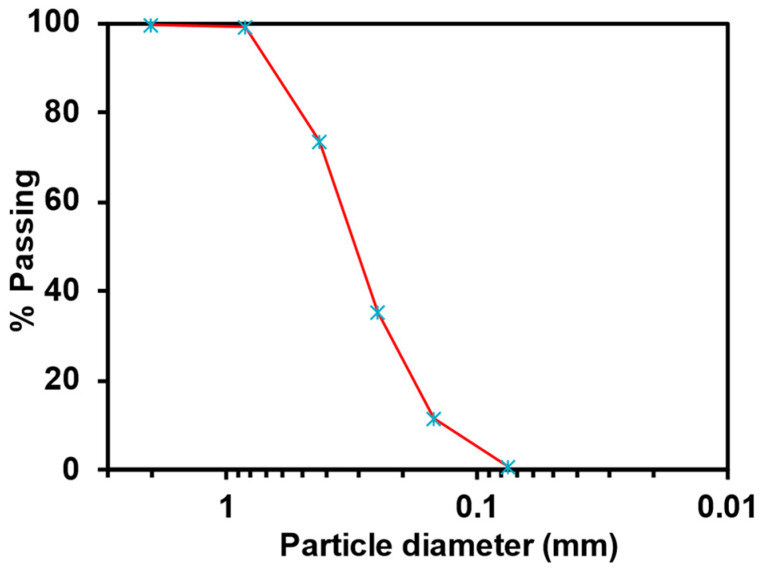
Particle-size distribution of the Thadiq sand.

The soil used in this study was obtained locally from Thadiq, located approximately 120 km north of Riyadh, Saudi Arabia. Several soil specimens were collected at depths ranging from 0–250 mm. Before use, the soil was air-dried in the laboratory and passed through a sieve with an opening diameter of 1.18 mm to remove the crop shaft, leaves, and sticks. Table 1 presents the basic properties of the sand; it is classified as poorly graded according to the Unified Soil Classification System. Figure 1 shows the particle-size distribution, with 99.2% sand and 0.8% fines.

### 2.2. Biopolymers

Experiments used acacia powder, pectin, and sodium alginate mixed with natural Thadiq sand to investigate the effects on the UCS measurements. The biopolymer was selected based on film formation, thickening, gelling capability, ease of use, availability, and sustainability.

Acacia gum, known as gum Arabic, is derived from exudates of mature Acacia senegal or Acacia seyal trees. The major components of this exudate are polysaccharides and glycoproteins [47]. Acacia gum is highly soluble in water and forms solutions with various concentrations, less viscous than solutions formed by other gums. It consists of D-galactose, D-glucuronic acid, arabinose, and rhamnose. The acacia used in this study was obtained from Qualikems Chem Pvt. It has a purity of 79.25%.

Sodium alginate is a hydrophilic and water-soluble anionic polysaccharide that can be extracted from the cell walls of various species of brown marine algae and the capsular component of bacteria such as Azotobacter vinelandii and Pseudomonas species [48,49]. It is comprised of (1,4)-β-D-mannuronate (M) and α-L-guluronate (G) residues arranged as M blocks, G blocks, and alternating M and G blocks [50]. The sodium alginate used in this study was obtained from Techno Pharmchem. It has a molecular weight of 216.121 g/mol and a purity of 57%.

Pectin is a significant component of the middle lamella of the primary cell wall and is mainly isolated commercially from citrus fruit peels and apple pomace cell walls [51]. Other sources of pectin include apricots, cherries, carrots, grapes, mangoes, pawpaw, and strawberries. It is a polysaccharide that contains significant amounts of galacturonic acid and smaller amounts of L-arabinose, D-galactose, and L-rhamnose. α-(1–4) linkages connect the galacturonic acid residues and form a linear chain. The pectin used in this study was obtained from Acros Organics.

### 2.3. Specimen Preparation

The biopolymers were mixed directly with the required amount of deionised water to form the treatment solutions with concentrations of 0%, 0.5%, 1%, 2%, 3%, and 5%. The slow addition of the biopolymer powder to the deionised water at the target concentration prevented clump formation. A high-speed mechanical stirrer was used to mix the solution for 10–15 min until the mixture became homogeneous. The ratio of the dry biopolymer weight to the total weight of the resulting treatment solution defined the target concentration.

Fabrication of UCS test specimens with a diameter of 49.80 mm and a height of 102.34 mm used a mould. The mixture had a moisture content of 10% and was compacted lightly with 25 blows and then compressed between two end plates using a hydraulic jack to achieve a wet density of 1.64 g/cm^3^. Specimens underwent different curing conditions before testing. 

The specimens used to determine the variation in the moisture of the biopolymer specimen with curing days were prepared in a container that had a diameter of 20 mm and a height of 30 mm. The specimens were compacted to achieve a wet density similar to the UCS. The resulting weight of the specimen was monitored as the curing proceeded over seven days. To ensure the repeatability of the test, three UCS samples were prepared for each test condition under consideration.

Unscathed chunks of the sand specimens collected from the UCS columns were examined via SEM to study the influence of the biopolymer. The piece was placed on a stopper and kept under vacuum conditions for 10 min, followed by the deposition of a platinum coating for 60 s to a thickness of 25 nm to prevent charging under the electron beam during the imaging process.

### 2.4. Unconfined Compressive Strength Tests (UCS)

UCS tests complied with ASTM D2166-06 (2007) [52], which specifies the standard test method for cohesive soil. The specimens were cured between 7 and 56 days at room temperature (25 °C) to examine the effect of biopolymer concentration on the UCS of the modified sand. Tests also investigated the effect of curing time on the UCS using stabilised sand specimens tested after curing periods of 0, 1, 3, 7, 14, 21, 28, and 56 days at room temperature (25 °C). Additionally, to evaluate the effect of the curing temperature (25, 40, 60, 85, and 110 °C) on the UCS, specimens were cured for 7, 14, 28, and 56 days. The specimens were allowed to cool for 15 min before testing. The test was conducted at an applied strain rate of 0.3 mm/min.

### 2.5. Scanning Electron Microscope Imaging

The surface morphology of the modified sand was characterised via field-emission SEM (JEOL; JSM7600F) together with energy-dispersive X-ray spectroscopy. For the SEM, electron beams were utilised to examine the surface morphology in detail. The main signals detected were from backscattered and secondary electrons, which generated a high-resolution image of the specimen.

## 3. Results

### 3.1. Effect of Biopolymer Concentration

**Figure 2:**
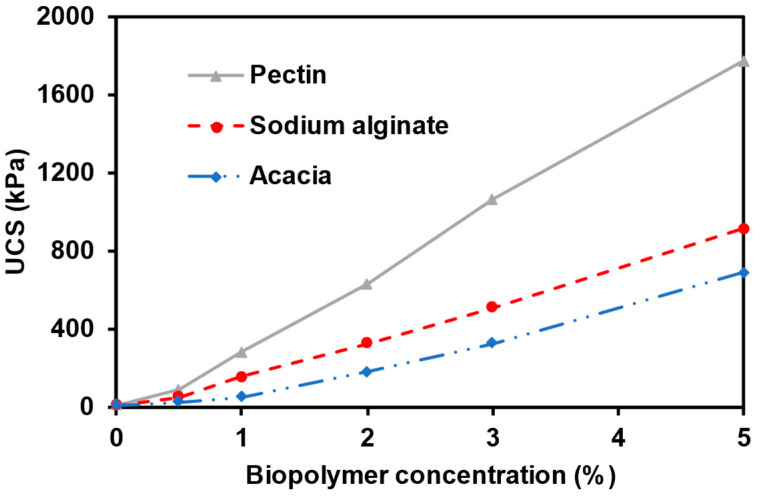
Effect of the biopolymer concentration on the UCS after a curing period of seven days.

Figure 2 presents the strength of Thadiq sand stabilised with the biopolymer at varying concentrations of 0%, 0.5%, 1%, 2%, 3%, and 5% after seven days of curing. The UCS increased significantly with an increase in the biopolymer concentration. There were insufficient bonds between the sand grains without adding the biopolymer (0% to represent the control specimen), resulting in a low compressive strength of 12.43 kPa after seven days. The obtained strength is due to compaction during specimen preparation. Incorporating 0.5% biopolymer concentration into the soil matrix improved the strength to 28.79 kPa, 54.66 kPa, and 92.38 kPa for acacia, sodium alginate, and pectin, respectively. However, the biopolymer concentration of 5.0% increases the compressive strength to 692.38 kPa, 916.68 kPa, and 1771.97 kPa for acacia, sodium alginate, and pectin, respectively. This increase in strength corresponds to a rise of approximately 54-, 71-, and 138-folds, respectively, compared to the control specimen. The results of this research complement the study by Fatehi et al. (2018) [39] and Fatehi et al. (2019) [38] utilising casein and sodium caseinate and sodium biopolymers, respectively. Ayeldeen et al. (2016) [34] made a similar observation with xanthan gum, guar gum, and modified starch on silty soil.

Remarkable improvement in strength with biopolymer concentration can be attributed to the increases in the viscosity and the binding capability of the biopolymers. Pectin biopolymer yielded the most significant improvement in the compressive strength of the Thadiq sand, followed by sodium alginate and acacia. The low strength of acacia-modified specimens can be attributed to the low viscosity in water compared to the other biopolymers. The low viscosity of acacia biopolymer results from the highly branched galactose/arabinose/rhamnose/glucuronic acid side chains attached to the galactan main chain [53]. The gelling property responsible for the strength of sodium alginate is a function of the ratio of β-D-mannuronate to α-L-guluronate units (M/G) within the structure. A lower M/G ratio corresponds to a stronger and more brittle gel [54]. α-L-guluronate units play a significant role in the gelation process, in contrast to β-D-mannuronate, which comprises alternating guluronate and mannuronate units. The linear sequence of (1,4)-α-D-galacturonic acid residues within the pectin structure forms aggregates held together by secondary valence bonds primarily responsible for the gelation property [55], which is responsible for the pectin strength.

**Figure 3:**
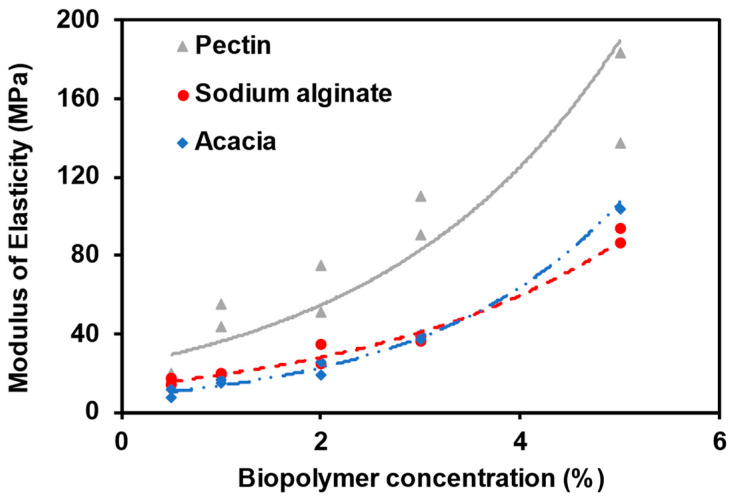
Effect of the biopolymer concentration on the stiffness of the modified sand after a curing period of seven days.

**Figure 4:**
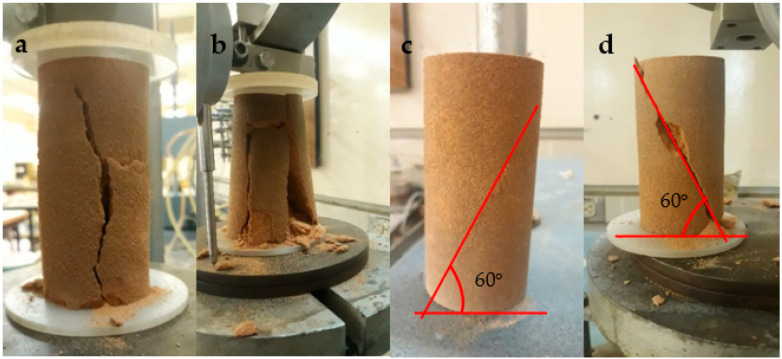
Failure planes for (**a**) control, (**b**) 5% Acacia, (**c**) 5% Sodium alginate, and (**d**) 5% Pectin, after a curing period of seven days.

Figure 3 presents the elastic modulus for the biopolymer-modified sand specimens to the concentration of the biopolymer after seven days of curing. The stiffness of the modified sand was determined in the inelastic region of the stress-strain curve. The secant modulus of elasticity, E50, was calculated from the slope of the line connecting 50% of the maximum UCS to the origin. It is defined as minor stress-dependent [56]. Results showed that increased biopolymer concentration increased the modulus of elasticity. Among the biopolymers, acacia powder yielded the least improvement in the compressive strength for all the concentrations tested; however, the stiffness at a concentration of 5% was higher than sodium alginate. Adding 5% pectin resulted in the most significant improvement in compressive strength and stiffness at the end of the 7-day curing period. These results agree with previous research by Ayeldeen et al. (2016) [34], which concluded that the elastic modulus increased as the biopolymer (xanthan gum, guar gum, or modified starch) concentration increased. Figure 4 shows the shear failure results for the control specimen and sand modified with acacia, sodium alginate, and pectin at a concentration of 5%. The failure planes of the control and acacia specimens were vertical, while that of the sodium alginate and pectin specimens were inclined at approximately 60°.

### 3.2. Effect of Curing Intervals

**Figure 5:**
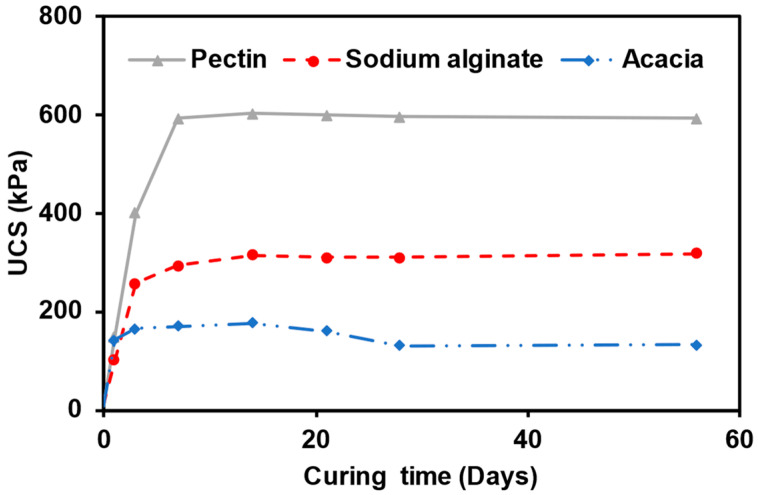
Effect of the curing time on the UCS of the modified sand.

The impact of varying curing time over 0, 1, 3, 7, 14, 21, 28, and 56 days was examined on the UCS of the biopolymer-modified sand at a room temperature of 25 °C. Since the UCS of the modified sand increases continually with the biopolymer concentration, a concentration of 2% was selected to study the effect of the curing time of the UCS. Figure 5 presents the influence of curing time on the UCS of sand modified with acacia, sodium alginate, and pectin biopolymer. When the modified sand was not allowed to cure and was tested immediately after preparation, a low UCS of 9.18 kPa, 12.11 kPa, and 9.66 kPa were obtained for acacia, sodium alginate, and pectin, respectively. The strength of the biopolymer-modified sand increased significantly after one day of curing to 142.31 kPa, 102.32 kPa, and 149.33 kPa for acacia, sodium alginate, and pectin, respectively. The results indicate that the curing time is an essential parameter for the strength development of biopolymer-modified sand.

The highest rate of strength increases in the biopolymer-modified sands occurs within the first seven days of curing. The significant effect of curing time on the strength development of the modified soil within the first seven days of curing agrees with previous studies by Latifi et al. (2017, 2016) [42,43]. This significant improvement in the strength was attributed to reduced moisture content within the specimens. After one day of curing, the modified specimen was observed to have achieved approximately 80, 32, and 25% of the maximum UCS achievable for acacia, sodium alginate, and pectin, respectively. All the biopolymers utilised reached maximum UCS gain after a curing period of 14 days. However, the UCS of specimens modified with acacia declined by 9% after 21 days and 8% after a curing period of 28 days. After that, the UCS maintained its value between 28 to 56 days of curing. However, the UCS of sodium alginate- and pectin-modified specimens remained relatively constant over the 14–56 days of curing.

**Figure 6:**
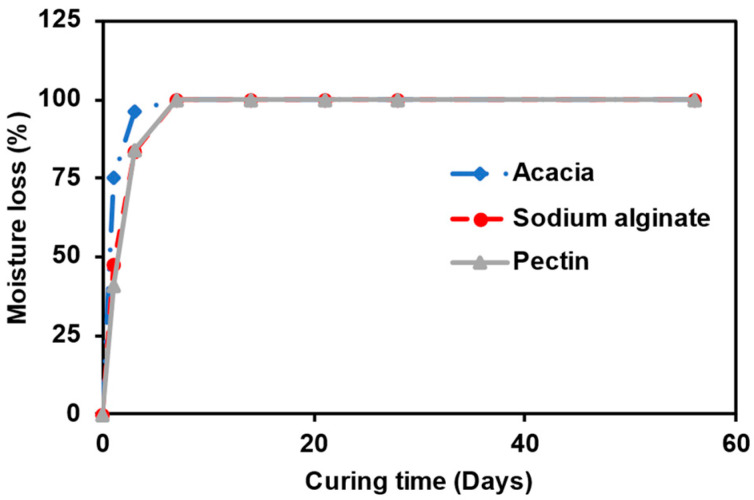
Variation of moisture loss with curing days for 2% biopolymer-modified sand.

As shown in Figure 6, the loss of moisture from the biopolymer-modified specimen played a crucial role in the rate of strength development. The rate of moisture loss was observed to be proportional to the increase in the UCS. The sodium alginate- and pectin-modified sand retained more moisture longer than the acacia-modified sand after one day of curing. The amount of water lost after one day of curing was 75, 47, and 41% for acacia, sodium alginate, and pectin, respectively. Furthermore, after three days of curing, the modified specimen was observed to have lost 96, 83, and 84%, respectively. The modified specimens were observed to lose all the moisture after seven days of curing completely. Furthermore, for both sodium alginate and pectin specimens, it was observed that the UCS decreased as the curing day was increased from 7 to 56 days, irrespective of the curing temperature.

### 3.3. Effect of Curing Temperature on UCS

**Figure 7:**
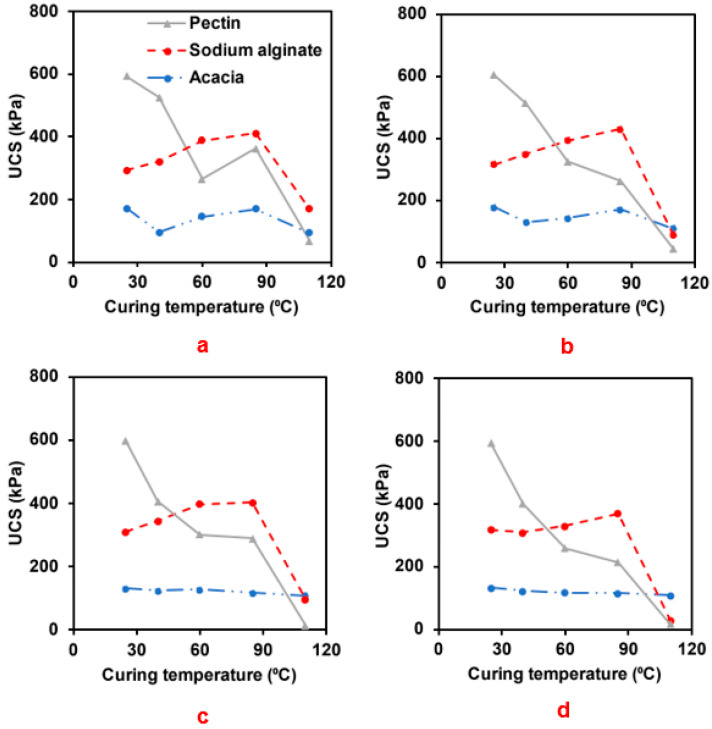
Effect of the curing temperature on the UCS of 2% biopolymer-modified sand at (**a**) 7 days, (**b**) 14 days, (**c**) 28 days, and (**d**) 56 days.

Figure 7 presents the results of the impact of curing temperature on the UCS of specimens modified with 2% biopolymer after curing periods of 7, 14, 28, and 56 days. Curing temperatures varied as 25, 40, 50, 85, and 110 °C. The strength development pattern of the modified specimen varied with the curing temperature. The UCS of the acacia-modified sand exhibited an erratic pattern as the curing temperature was increased from 25 to 110 °C. However, it is fair to conclude that increasing the curing temperature from 25 to 110 °C did not have a significant impact on the UCS compared to the other biopolymers. The UCS of the sodium alginate-modified sand increased as the curing temperature rose to 85 °C. A further increase in the temperature to 110 °C resulted in a significant reduction in the UCS. This strength reduction can be attributed to the thermal decomposition of the sodium alginate—biopolymer bond between adjacent soil grains. The thermal decomposition resulted in a breakdown of the linear polymer α-L-guluronic (G) acid residue responsible for gelation within the structure. The thermal decomposition of sodium alginate is due to dehydration at 103 °C, the destruction of glycosidic bonds at 212 °C, and the conversion of alginate to carbonaceous residue [57]. This finding contradicts the finding reported by Fatehi et al. (2019) [38], where UCS was reported to increase with curing temperatures up to 45 °C with further increase in the temperature resulting in a reduction in UCS. This discrepancy in results may result from the type of sodium alginate used. In contrast, the UCS of the pectin-modified sand decreased significantly as the curing temperature was increased from 25 to 110 °C. The reduction in the UCS can mainly be ascribed to the breakdown of primary chemical valence linkages between the galacturonic acid polymeric units of the pectin biopolymer network within the soil matrix.

For instance, at 56 days, the UCS of the acacia-modified specimen decreased by 8%, 11%, 13%, and 17%; the pectin-modified specimen decreased by 33%, 56%, 64%, and 97%, and the sodium-alginate modified specimen increased by −3, 4, 16, and −91% as the curing temperature was varied from 25 °C to 40 °C, 60 °C, 85 °C, and 110 °C, respectively.

### 3.4. Effect of Moisture Loss on UCS 

**Figure 8:**
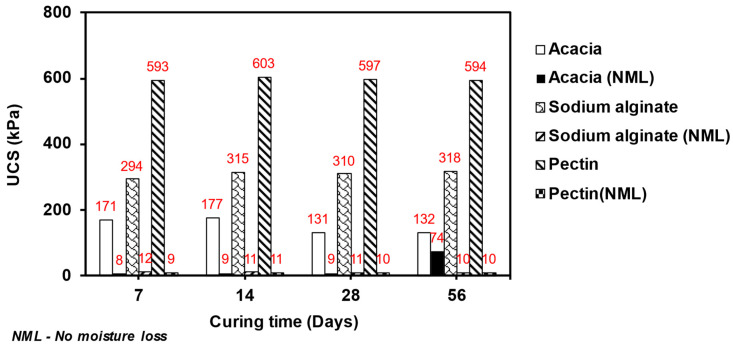
Effect of moisture loss on the UCS of the 2% biopolymer-modified sand.

Moisture loss significantly resulted in the strength development of the biopolymer-modified sand. Therefore, additional analyses were conducted to further examine the impact of preventing moisture loss with curing days on the strength. Figure 8 presents the effect of no moisture loss on the UCS of sand treated with 2% biopolymer concentration and cured for 0, 7, 4, 28, and 56 days. After seven days of curing, the UCS was 8.16 kPa, 12.17 kPa, and 9.02 kPa for the sand modified with acacia, sodium alginate, and pectin, respectively. However, when the specimens were allowed to lose moisture, the UCS rose to 171.46 kPa, 293.57 kPa, and 592.62 kPa, respectively. As the curing time increased from 0 to 56 days, the strength of the acacia-modified specimens increased significantly to 74 kPa, while the other two biopolymer-modified specimens both maintained a low strength of approximately 10 kPa, respectively. The acacia-modified specimen lost much moisture over the 56-day curing period. The moisture loss was faster than the other biopolymer-modified specimens owing to their low viscosity. Thus, moisture loss significantly affects the UCS of biopolymer-modified sand.

**Figure 9:**
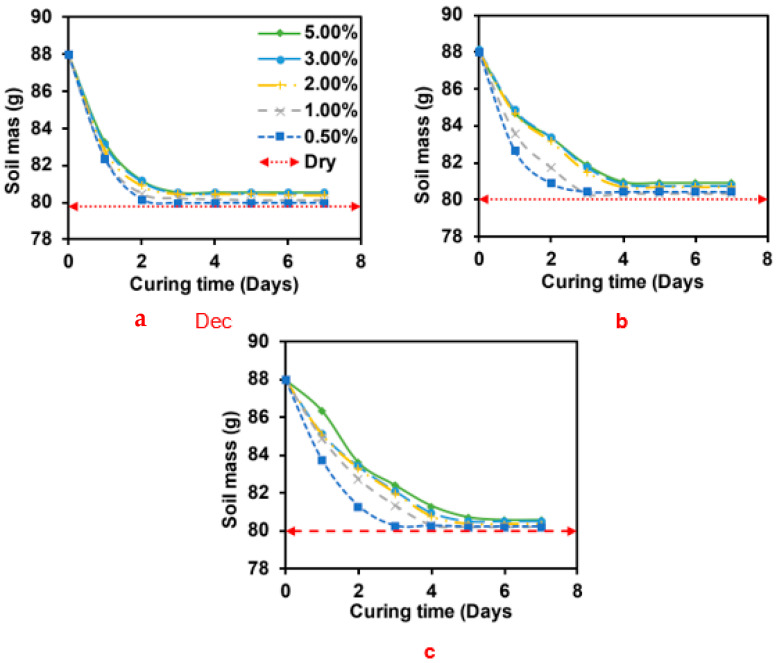
Variation of moisture content of soil specimen modified with 2% concentration of (**a**) Acacia, (**b**) Sodium alginate, and (**c**) Pectin.

Furthermore, an in-depth investigation was conducted to monitor the moisture loss from the biopolymer-modified specimens cured under laboratory conditions. The result is illustrated in Figure 9. The time required by the biopolymer-modified specimen to reach complete dehydration depend on the type and concentration of the biopolymer used. The acacia-modified specimens were observed to achieve complete dehydration after three days of curing across all biopolymer concentrations. However, sodium alginate-modified specimens with a concentration of 0.5% and 1% required three days, 2% took four curing days, and those with 3% and 5% took five curing days to complete dehydration. The pectin-modified specimens required 3, 5, and 6 curing days at a biopolymer concentration of 0.5%, 1–3%, and 5%, respectively.

### 3.5. Microstructure Scaling Analysis

**Figure 10:**
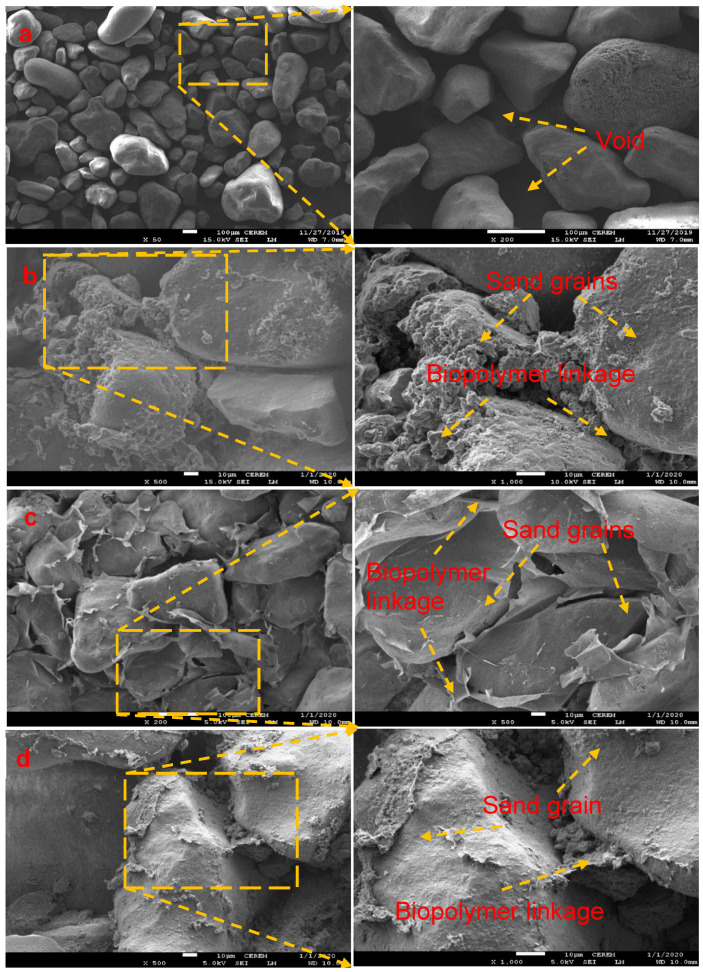
SEM images of (**a**) natural sand; (**b**) 2% Acacia-modified; (**c**) 2% Sodium alginate-modified; (**d**) 2% Pectin-modified.

Figure 10a–d present the micrographs of Thadiq sand in its natural state and a biopolymer-modified specimen after UCS testing. Images of the natural sand display dispersed grains of sand with noticeable pockets of voids and pores, as shown in Figure 10a. The soil particles had no significant link, resulting in a low UCS. Figure 10b–d shows the micrographs of the specimens modified with a biopolymer concentration of 2.0%. Accumulation of the biopolymer between the sand particles was observed, which was responsible for the binding effect within the matrix of the modified sand. Thus, the UCS of the modified sand is dependent on the strength of the biopolymer linkages or the binding force of the sand grains. Generally, the biopolymer stabilization’s reinforcement mechanism depends on moisture loss from the biopolymer-sand admixture. The mixture of biopolymer with water results in the formation of a hydrogel absorbed onto the surface of the sand particles via hydrogen and intermolecular bond. The hydrogel becomes a thin and hardened membrane network as the moisture evaporates, thus increasing strength and elasticity modulus. As a result, with more biopolymer concentration, the polymer membrane gets harder, which results in more stiffness.

**Figure 11:**
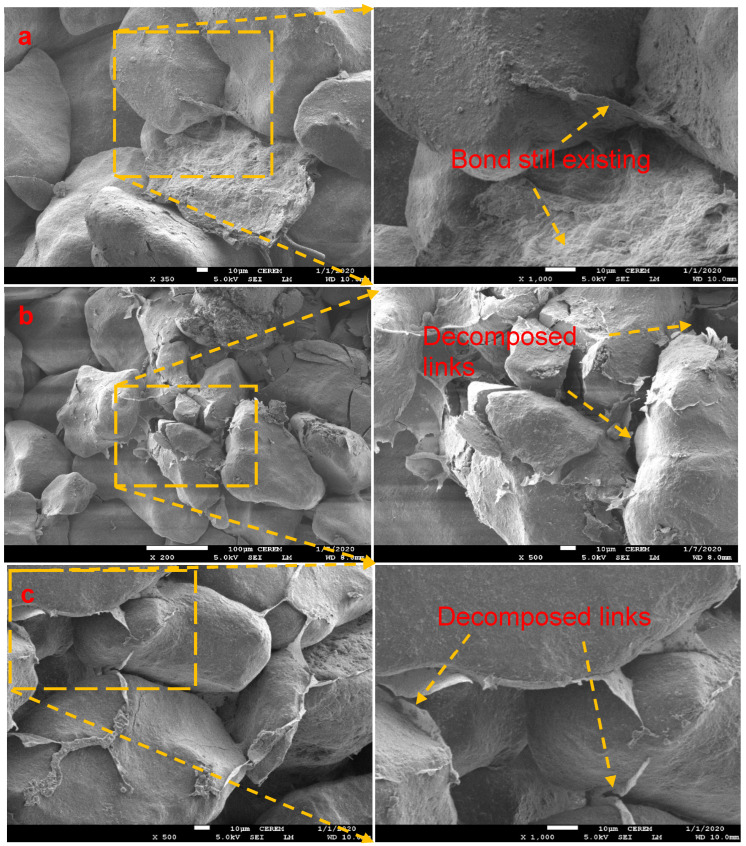
SEM images of modified specimens after curing at 110 °C for 2% (**a**) Acacia, (**b**) Sodium alginate, and (**c**) Pectin.

Figure 11a–c presents micrographs of the biopolymer-modified sand subjected to an elevated temperature of 110 °C for 56 days after UCS testing. As shown in Figure 11a, the acacia biopolymer links between the sand particles were not significantly affected by the temperature. This signifies why there were no significant changes in the UCS values obtained after curing at 7, 14, 28 and 56 days. However, Figure 11b,c show the scanty sight of sodium alginate and pectin biopolymer films observed on the surface and within the pore spaces of the sand grains. The links between the sand grains were observed to be decomposed after exposure to a temperature of 110 °C, leaving thin biopolymer film residues around the sand grains, which resulted in a reduction in the UCS compared to the specimens cured at a room temperature of 25 °C.

## 4. Conclusions

This research compared the effects of acacia, sodium alginate, and pectin on the soil properties of Thadiq dune sand. A control specimen and three biopolymer-modified sand specimens were tested at different conditions, including five concentrations, curing periods, and temperature, to examine these effects on the UCS and shear wave velocity. Salient conclusions are below:

- Incorporating the biopolymers within the sand matrix improved the strength and stiffness of the sand. An increase in the biopolymer concentration increased the strength and stiffness.
- Pectin yielded more significant improvements in the strength and stiffness of the sand compared to the acacia and sodium alginate biopolymers.
- The increased strength of the modified sand significantly improved within the first seven days of curing compared with the more extended curing period from 7 to 56 days, in which the increase in strength was minor.
- Moisture loss over time significantly affected the strength of the biopolymer-modified sand. UCS results for specimens protected against moisture loss indicated that the strength remained relatively constant as the curing time increased from 0 to 56 days, except for the acacia-modified sand, whose strength increased significantly after 56 days.
- The temperature significantly affected the strength of the specimens. The acacia-modified sand exhibited an irregular strength development pattern, with optimum results obtained at room temperature. The optimum curing temperature for the sodium alginate-modified sand was 85 °C; higher temperatures had detrimental effects on strength.
- SEM images indicated that incorporating the biopolymer within the sand matrix led to the formation of a bond between the soil particles, which enhanced the strength and stiffness of the Thadiq sand. The SEM images indicated that cementation occurred even when the acacia-modified sand was subjected to a high temperature of 110 °C. In contrast, the films on the surface and within the grains were mostly decomposed for the pectin-modified sand.
- The durability of biopolymers is a major concern. Hence, further studies should be conducted on repeated wet-dry cycles and other environmental conditions.

## Figures and Tables

**Table 1:** Basic properties of the Thadiq sand.

Specific Gravity, Gs	e_min_	e_max_	D50 (mm)	D10 (mm)	Coefficient of Uniformity, Cu	Coefficient of Curvature, Cc	pH
2.66	0.51	0.76	0.15	0.09	1.99	0.88	8.33

## Data Availability

All data related to this manuscript is available upon request.
